# Supplementary material for: Investigating the influence of physiologically relevant hydrostatic pressure on CHO cell batch culture
Source: Sci Rep. 2021 Jan 8;11:162. doi: 10.1038/s41598-020-80576-8 (PMC7794228; doi:10.1038/s41598-020-80576-8)
Supplement: Supplementary file 1 — Supplementary Information 1. [file 41598_2020_80576_MOESM1_ESM.zip › S1/IgG/Jan17 standard curve/2/8.pdf]

=====================================================================

Acq. Operator : SYSTEM Seq. Line : 6
Sample Operator : SYSTEM
Acq. Instrument : HPLC-0039 Location : 2
Injection Date : 1/17/2019 3:22:45 PM Inj : 1
Inj Volume : 100.000 µl
Different Inj Volume from Sample Entry! Actual Inj Volume : 80.000 µl
Sequence File : C:\Users\Public\Documents\ChemStation\1\Data\TK_CHO_IgG 2019-01-17 15-00-19\TK_CHO_IgG.S
Method : C:\Users\Public\Documents\ChemStation\1\Data\TK_CHO_IgG 2019-01-17 15-00-19\CHO_IgG_LC.M (Sequence Method)
Last changed : 12/4/2018 1:38:41 PM by SYSTEM

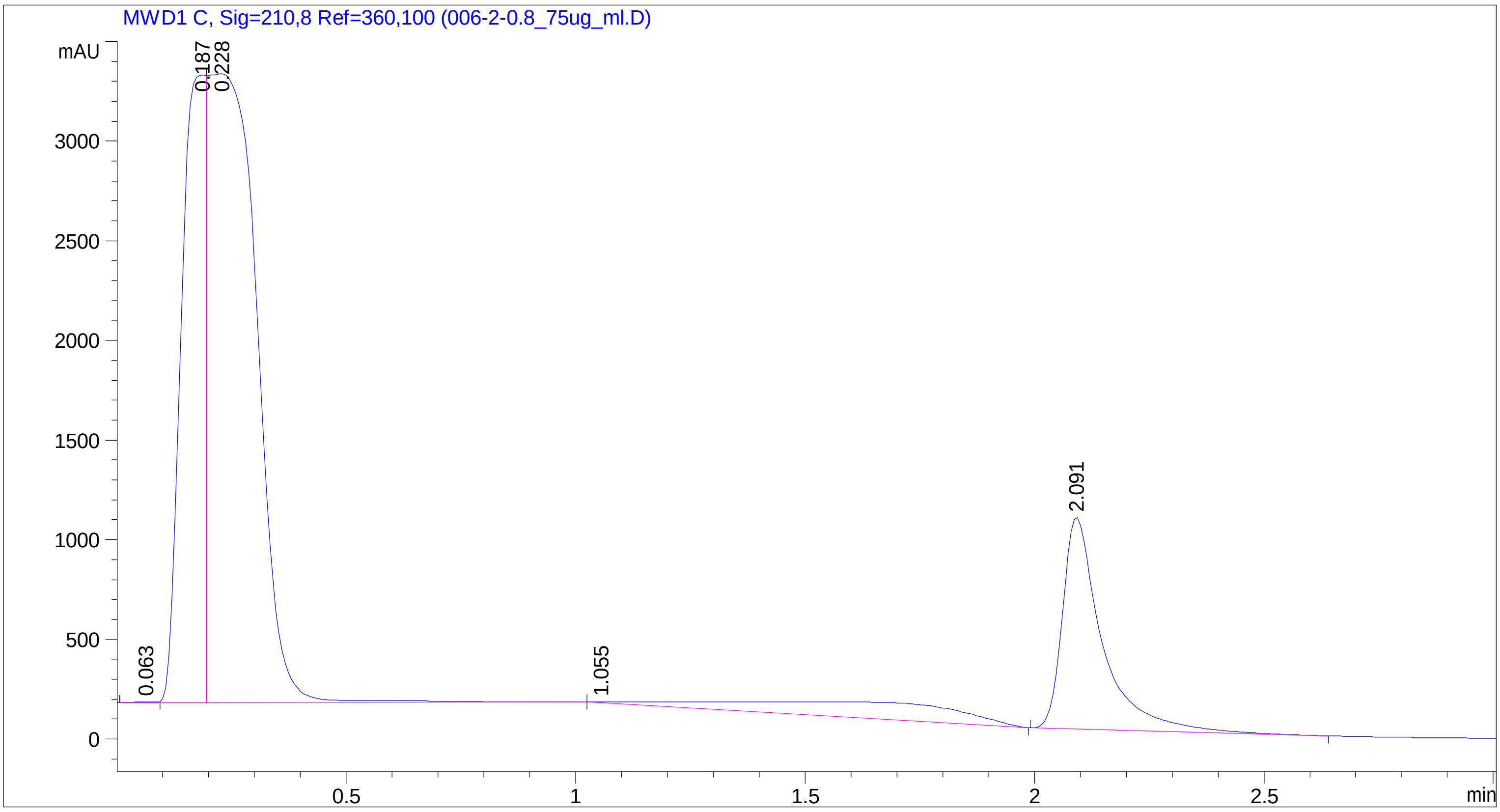


=====================================================================

Area Percent Report

=====================================================================

Sorted By : Signal
Multiplier : 1.0000
Dilution : 1.0000
Use Multiplier & Dilution Factor with ISTDs

Signal 1: MWD1 C, Sig=210,8 Ref=360,100

| Peak # | RetTime [min] | Type | Width [min] | Area [mAU*s] | Height [mAU] | Area % |
|---|---|---|---|---|---|---|
| 1 | 0.063 | BV E | 0.0399 | 9.29272 | 3.62937 | 0.0214 |
| 2 | 0.187 | VV R | 0.0582 | 1.12693e4 | 3147.43970 | 25.9122 |
| 3 | 0.228 | VB | 0.1134 | 2.30859e4 | 3153.43359 | 53.0829 |
| 4 | 1.055 | BB | 7.6678 | 2730.63306 | 4.16014 | 6.2787 |
| 5 | 2.091 | BBA | 0.0882 | 6395.19385 | 1062.56519 | 14.7049 |

| | | |
|---|---|---|
| Totals : | 4.34903e4 | 7371.22799 |

====================================================================

*** End of Report ***
